# Ferroptosis in a sarcopenia model of senescence accelerated mouse prone 8 (SAMP8)

**DOI:** 10.7150/ijbs.53126

**Published:** 2021-01-01

**Authors:** Yan Huang, Beiling Wu, Dingzhu Shen, Jiulin Chen, Zhihua Yu, Chuan Chen

**Affiliations:** Shanghai Geriatric Institute of Chinese Medicine, Shanghai University of Traditional Chinese Medicine, Shanghai 200031, People's Republic of China.

**Keywords:** Sarcopenia, Ferroptosis, Iron overload, C2C12, SAMP8

## Abstract

As a systemic syndrome characterized by age-associated degenerative skeletal muscle atrophy, sarcopenia leads to a risk of adverse outcomes in the elderly. Age-related iron accumulation is found in the muscles of sarcopenia animal models and patients, but the role of iron in sarcopenia remains poorly understood. It has been recently found that iron overload in several diseases is involved in ferroptosis, an iron- dependent form of programmed cell death. However, whether this excess iron can result in ferroptosis in muscles is still unclear. In our present study, we found that ferric citrate induced ferroptosis in C2C12 cells, as well as impaired their differentiation from myoblasts to myotubes. Due to the decreased muscle mass and fiber size, 40-week-old senescence accelerated mouse prone 8 (SAMP8) mice were used as a sarcopenia model, in whose muscles the iron content and markers of ferroptosis were found to increase, compared to 8-week- old SAMP8 controls. Moreover, our results showed that iron overload upregulated the expression of P53, which subsequently repressed the protein level of Slc7a11 (solute carrier family 7, member 11), a known ferroptosis-related gene. The downregulation of Slc7a11 then induced the ferroptosis of muscle cells through the accumulation of lipid peroxidation products, which may be one of the causes of sarcopenia. The findings in this study indicate that iron plays a key role in triggering P53- Slc7a11-mediated ferroptosis in muscles, and suggest that targeting iron accumulation and ferroptosis might be a therapeutic strategy for treating sarcopenia.

## Introduction

Sarcopenia, recently defined by the World Health Organization as a disease 1, is characterized by the age-associated degenerative loss of skeletal muscle strength and mass 2. As a systemic syndrome with progressive weakness and wasting of muscles, sarcopenia contributes to the risk of adverse outcomes in the elderly, such as loss of autonomy, physical disability, and even death, which also has a series of economic and social implications 3, including increasing healthcare costs and impaired dignity of the aged 4. It is believed that the development of sarcopenia is associated with age, nutrition, physical exercise, and other diseases, in addition to mitochondrial dysfunction, oxidative stress, inflammation, and many other pathways, which have also been reported to be involved 5. Despite many different strategies based on these pathophysiological mechanisms having been proposed to prevent muscle atrophy 6, no potential pharmacological approaches are currently in clinical use 7, suggesting that the pathogenesis of sarcopenia is worthy of further scrutiny.

Iron is an essential trace element required for the survival and health maintenance of all cells and organs. It plays important role in many biological functions, such as erythrocyte oxygen transport, respiratory complex regulation, DNA synthesis, ATP synthesis, and systemic immune responses 8-10. However, iron accumulation has been confirmed to disrupt normal cellular function via oxidative stress and the catalysis of highly toxic hydroxyl-radicals 11, which then cause various disorders via injury to DNA, lipids, and proteins and lead to multiple diseases involving the nervous, cardiovascular, liver, and musculoskeletal systems 10. As the organ that contains 10%-15% of the body's total iron content, skeletal muscle is expected to be impaired due to iron disorders. In recent studies, age-related iron overload in skeletal muscles has been found and demonstrated to induce muscle atrophy or dysfunction, suggesting that sarcopenia might be associated with iron accumulation 12-14.

Ferroptosis is a recently discovered form of cell death, found in some tumor cells treated with the oncogenic RAS-selective lethal small molecule erastin 15. It is characterized as an iron- dependent cell death related to the requirement for redox active iron 15, which is also morphologically, biochemically, and genetically different from other forms of cell death such as necrosis, apoptosis, or autophagy 16, 17. In addition, ferroptosis can be inhibited by the small molecule ferrostatin-1 or the deferoxamine (DFO, a chelator of intracellular iron) but not by other inhibitors of other forms of cell death 18, 19. The induction of ferroptosis involves various events including the activation of mitochondrial voltage-dependent anion channels and mitogen-activated protein kinases, the upregulation of endoplasmic reticulum stress, and the inhibition of a cystine/glutamate antiporter, which finally results in the accumulation of lipid peroxidation products 20. An increasing number of studies have demonstrated that ferroptosis contributes to physiological and pathological processes in multiple diseases, including cancers 21, neurodegenerative diseases 22, hepatic and heart ischemia/reperfusion injury 19, and acute renal failure 23. Moreover, it has been found that iron overload can induce ferroptsis in hemochromatosis and cardiomyopathy 24, 25. However, the precise roles of iron metabolism and ferroptosis in sarcopenia are currently unknown.

In this study, we studied iron overload-treated C2C12 cells and muscle tissue from senescence accelerated mouse prone 8 (SAMP8) mice that have been shown to be a relevant sarcopenia model 26, 27, and investigated the role of iron overload in triggering ferroptosis in muscles, which might further elaborate the regulation of iron metabolism in sarcopenia.

## Materials and methods

### Cell culture and differentiation

C2C12 myoblasts (Cell Bank, Shanghai Institute for Biological Science, Shanghai, China) were cultured in the growth medium (GM), comprising Dulbecco's modified Eagle's medium (DMEM, 25 mM glucose, Thermo Fisher Scientific, Waltham, MA, USA) supplemented with 10% fetal bovine serum (FBS, Thermo Fisher Scientific, Waltham, MA, USA) and 1% penicillin-streptomycin (Thermo Fisher Scientific, Waltham, MA, USA). When cells reached 80% confluence, the medium was switched to differentiation medium (DM), consisting of DMEM supplemented 2% horse serum (Thermo Fisher Scientific, Waltham, MA, USA) and 1% penicillin‑streptomycin. Five days after differentiation, cells were used for experiments. All cells were cultured in an incubator at 37ºC in a humidified atmosphere of 95% air and 5% CO_2_, and the culture medium was changed every other day.

### Mice

The study was approved by the Animal Care Ethics Committee of the Use of Laboratory Animals at Shanghai University of Traditional Chinese Medicine. The 8-week- and 40-week-old male SAMP8 mice were purchased from the model animal research center of Zhishan Institute of Healthcare Research Co., Ltd. (Beijing, China). All the mice were kept in an SPF grade animal facility at 24°C with a relative humidity of 50%-60%, and in a light/dark cycle of 12 h/12 h.

### Cell treatments

Ferric citrate (FAC) was dissolved in sterile deionized water and used at indicated concentrations. Erastin and ferrostatin-1 were dissolved in dimethylsulfoxide (DMSO) to a working concentration of 1 μM and 10 μM, respectively. Deferoxamine (DFO) was dissolved in sterile deionized water and used at a concentration of 100 μM. All reagents were purchased from Sigma-Aldrich, St. Louis, MO, USA. For various tests, C2C12 myoblasts and myotubes were treated for 48 h, and differentiating C2C12 cells were treated for 5 days.

### Cell viability assay

Cell viability was measured using the AlamarBlue Cell Viability Reagent (Invitrogen, Carlsbard, CA, USA) according to the manufacturer's instructions. In brief, after cells were cultured in medium supplemented with 10% alamarBlue for 2 h, 100 μl of the culture medium was transferred to 96-well plates and quantified spectrophotometrically with a microplate reader (SpectraMax i3x, Molecular Devices, CA, USA) at wavelengths of 570 nm and 650 nm. The culture medium from no cell-seeded disks was similarly analyzed as blank controls.

### Lipid peroxidation measurements

Intracellular lipid peroxidation was evaluated with the BODIPY lipid probe C11 (581/591) (Invitrogen, Carlsbard, CA, USA). After treatment, cells were stained with C11- BODIPY fluorescent dye in the dark at 37°C for 30 min. For fluorescence measurements, the cells were washed twice with PBS and analyzed by a microplate reader (SpectraMax i3x, Molecular Devices, CA, USA). For fluorescence imaging, cells were then stained by Hoechst (Beyotime, Shanghai, China) for 5 min and detected by a fluorescence microscope (CX43 Biological Microscope, Olympus, Tokyo, Japan).

### Iron parameters

Tissue non-heme iron was measured using a colorimetric Iron Assay kit (Abcam, Cambridge, UK). Following the manufacturer's instructions, tissue was homogenized in Iron Assay Buffer on ice and centrifuged at 16,000 *g* for 10 min to collect the supernatant. The supernatant was then mixed with Iron Reducer in a 96-well plate and incubated at 37°C for 30 min. Subsequently, the Iron Probe was added, mixed, and incubated at 37°C for 60 min protected from light, and the plate was then measured immediately at 593 nm on a colorimetric microplate reader (SpectraMax i3x, Molecular Devices, CA, USA).

### NADPH assay

Nicotinamide adenine dinucleotide phosphate (NADPH) content was measured using a fluorometric NADP/NADPH Assay Kit (Abcam, Cambridge, UK). Cells or tissues were homogenized in Lysis Buffer, and the homogenate was then centrifuged at 2,500 *g* for 5 min. The collected supernatant was mixed with NADPH Extraction Solution in a 96-well plate and incubated at room temperature for 15 min, before being neutralized with NADP Extraction Solution. The NADPH Reaction Mixture was then added, mixed, and incubated at room temperature for 2 h in the dark, and the fluorescence was immediately measured at Ex/Em = 540/590 nm on a microplate reader (SpectraMax i3x, Molecular Devices, CA, USA).

### Measurement of MDA and GSH content

The levels of malondialdehyde (MDA) and glutathione (GSH) in cells and tissues were detected using a Lipid Peroxidation MDA Assay Kit (Beyotime, Shanghai, China) and a GSH and GSSG Assay Kit (Beyotime, Shanghai, China), respectively. Both measurements strictly followed the manufacturer's instructions and the contents are indicated as μmol/g protein.

### Quantitative real-time PCR analysis

Total RNA was isolated from cells and tissues using TRIZOL reagent (Invitrogen, Carlsbard, CA, USA). An equivalent amount of each RNA sample was converted to cDNA using the PrimeScript RT kit (Takara, Shiga, Japan). PCR amplification was performed on a real-time PCR machine (ABI 7500, Thermo Fisher Scientific, Waltham, MA, USA) using a SYBR Green PCR kit (SYBR Premix EX Taq, TaKaRa, Shiga, Japan) with GAPDH (glyceraldehyde-3-phosphate dehydrogenase) used for normalization. The data were analyzed using the 2^-ΔΔCt^ method. The primers for RT-qPCR are listed below:

- Mouse *Ptgs2*-F: 5'- CTGCGCCTTTTCAAGGATGG-3',
- Mouse *Ptgs2*-R: 5'- GGGGATACACCTCTCCACCA-3',
- Mouse *Fbxo32*-F: 5'-AGAGAGGCAGATTCGCAAGCGT-3',
- Mouse *Fbxo32*-R: 5'-TGCAAAGCTGCAGGGTGACCC-3',
- Mouse *Trim63*-F: 5'-ACCTGCTGGTGGAAAACATC-3',
- Mouse *Trim63*-R: 5'-CTTCGTGTTCCTTGCACATC-3',
- Mouse *Il1b*-F: 5'-GAAATGCCACCTTTTGACAGTG-3',
- Mouse *Il1b*-R: 5'-TGGATGCTCTCATCAGGACAG-3',
- Mouse *Tnfa*-F: 5'- CCCTCACACTCAGATCATCTTCT-3',
- Mouse *Tnfa*-R: 5'-GCTACGACGTGGGCTACAG-3',
- Mouse *Col1a1*-F: 5'-GCTCCTCTTAGGGGCCACT-3',
- Mouse *Col1a1*-R: 5'-CCACGTCTCACCATTGGGG-3',
- Mouse *Col3a1*-F: 5'-CTGTAACATGGAAACTGGGGAAA-3',
- Mouse *Col3a1*-R: 5'-CCATAGCTGAACTGAAAACCACC-3.

### Transmission electron microscopy

Blocks smaller than 2 mm^3^ of harvested tissue were fixed in 2% osmium tetroxide for 2 h, washed several times with PBS, dehydrated in graded concentrations of ethanol, embedded in SPI-Pon 812 Epoxy Resin (Sigma-Aldrich, St. Louis, MO, USA) and polymerized for 48 h at 60°C. Sections were cut with a diamond knife (Daitome, Nidau, Switzerland) using a Leica Ultramicrotome (EM UC7, Leica, Hamburg, Germany) and stained with both methanolic uranyl acetate and lead citrate before viewing in a Tecnai G2 20 Twin (FEI, Thermo Fisher Scientific, Waltham, MA, USA) transmission electron microscope.

### Western blot analysis

Cells were lysed in radio immunoprecipitation assay (RIPA) lysis buffer with protease inhibitor cocktail for 30 min on ice. Then the lysates were centrifuged at 12,000 g for 10 min, and the protein in the supernatant was collected. Protein concentrations were measured by performing a bicinchoninic (BCA) assay. Each protein lysate (20 μg) was resolved by sodium dodecyl sulfatepolyacrylamide gel electrophoresis (SDS-PAGE) using 10% gels and proteins were then transferred to polyvinylidene difluoride (PVDF) membranes (Millipore, Burlington, MA, USA). Nonspecific interactions were blocked with 5% skim milk for 1 h, and membranes were then probed with specific primary antibodies to SLC7A11 (1:1000, Abcam, Cambridge, UK), Gpx4 (1:1000, Abcam, Cambridge, UK), P53 (1:1000, Cell Signaling Technology, MA, USA) P21 (1:1000, Cell Signaling Technology, MA, USA), and GAPDH (1:1000, Cell Signaling Technology, Danvers, MA, USA) overnight at 4ºC. HRP-conjugated secondary antibodies (1:3000, Genscript, Jiangsu, China) were used at 37°C for 1 h. The bound antibodies were visualized using an enhanced chemiluminescence detection system (Millipore, Burlington, MA, USA) and quantified using Image-Pro Plus 6.0 software (Media Cybernetics, MD, USA).

### Immunological staining and histomorphometric analysis

Tissues were fixed in 4% paraformaldehyde for 24 h, embedded in paraffin, and sectioned at a thickness of 5 μm. The sections for immunohistochemical staining were incubated with primary antibody to 4-Hydroxynonenal (1:200, Abcam, Cambridge, UK) overnight at 4ºC and secondary anti-goat IgG (Sigma-Aldrich, St. Louis, MO, USA) for 30 min at room temperature. A streptavidin-horse radish peroxidase (HRP) detection system was used to detect the antigen and sections were visualized with diaminobenzidine tetrahydrochloride. For immunofluorescence, sections were processed using rabbit anti-mouse LaminA primary antibody (1:200, Sigma-Aldrich, St. Louis, MO, USA) or rabbit anti-mouse xCT primary antibody (1:200, Abcam, Cambridge, UK), and Cy3- or Alexa Fluor 488-labeled anti-mouse IgG antibody (both from R&D Systems, Minneapolis, MN, USA) and DAPI (1 mg/ml, Roche, Basel, Switzerland). Stained sections were visualized using a fluorescence microscope (CX43 Biological Microscope, Olympus, Tokyo, Japan) and analyzed using Image-Pro Plus 6.0 software (Media Cybernetics, Rockville, MD, USA).

### Statistical analysis

All data were obtained from three or more experiments, and values are presented as means ± standard deviation. Statistical analyses were performed using a one-way analysis of variance (ANOVA), followed by the Student-Newman-Keul post hoc test. *P* < 0.01 was considered to indicate a statistically significant difference.

## Results

### Iron overload induces ferroptosis in C2C12 myoblasts and myotubes

**Figure 1:**
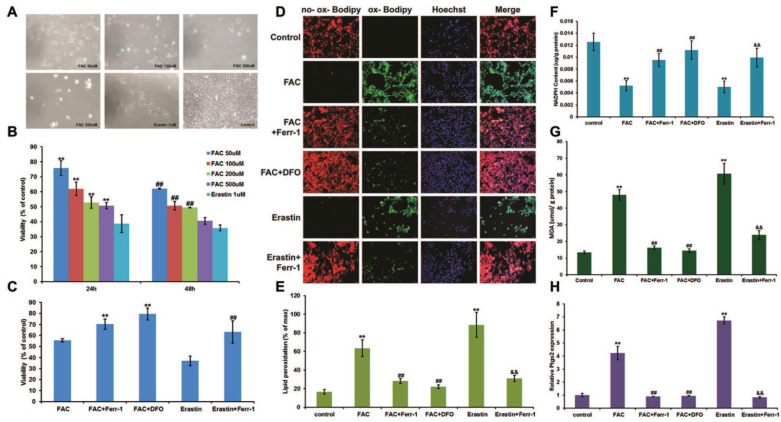
Ferroptosis in C2C12 myoblasts. (**A**) Morphology of C2C12 myoblasts treated with different concentrations of FAC for 24 h. (Original magnification: 200 x) (**B**) Cell viability with different concentrations of FAC for 24 h and 48 h. **P < 0.01 versus 1mM erastin for 24 h and ^##^*P* < 0.01 versus 1mM erastin for 48 h (**C**) Cell viability with 500 µM FAC or 1 µM erastin with or without inhibitors (ferr-1 or DFO) for 48 h. **P < 0.01 versus FAC and ^##^*P* < 0.01 versus erastin. (**D**) C2C12 myoblasts stained by C11-BODIPY after 24 h treatment (red: no probe staining, and green: ox-probe staining. Original magnification: 200 x). Lipid peroxidation (**E**), NADPH (**F**), MDA content (**G**), and *Ptgs2* mRNA level (**H**) in C2C12 myoblasts treated with 500 µM FAC or 1 µM erastin with or without inhibitors (ferr-1 or DFO) for 24 h. ^**^P < 0.01 versus control, ^##^*P* < 0.01 versus FAC, and ^&&^*P* < 0.01 versus erastin.

**Figure 2:**
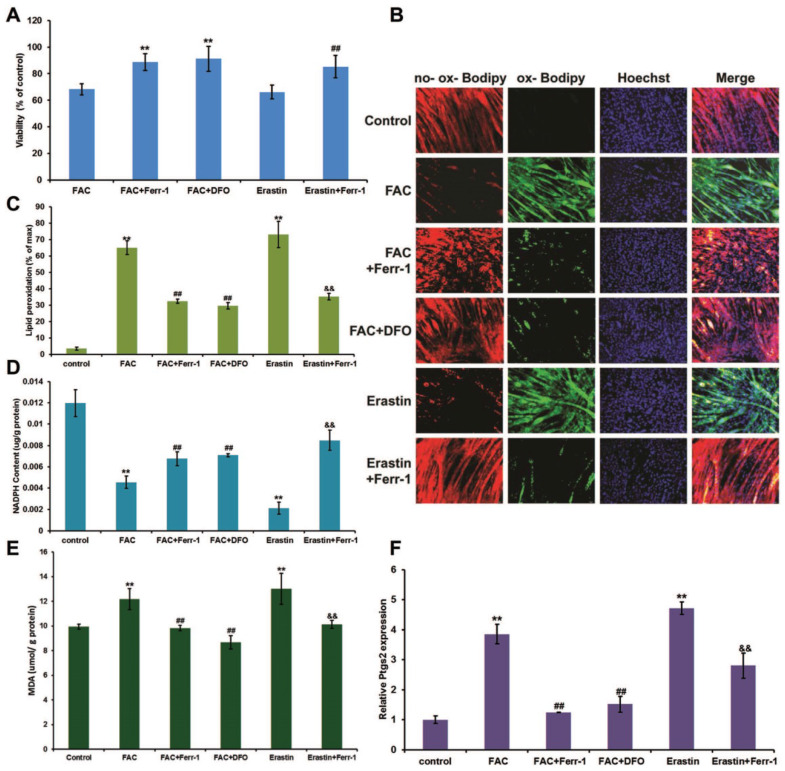
Ferroptosis in C2C12 myotubes. Differentiated C2C12 myotubes were treated with 1 mM FAC or 1 µM erastin with or without inhibitors (ferr-1 or DFO). (**A**) Cell viability after 48 h treatment. ^**^P < 0.01 versus FAC, and ^##^*P* < 0.01 versus erastin. (**B**) Lipid peroxidation of C2C12 myotubes stained with C11-BODIPY after 24 h treatment (red: no probe staining, and green: ox-probe staining. Original magnification: 200 x). Lipid peroxidation (**C**), NADPH (**D**), MDA content (**E**), and *Ptgs2* mRNA level (**F**) in C2C12 myotubes after 24 h treatment.^ **^P < 0.01 versus control, ^##^*P* < 0.01 versus FAC, and^ &&^*P* < 0.01 versus erastin.

To determine the proper concentration of FAC, a gradient of dilutions was used (50 μM, 100 μM, 200 μM, and 500 μM) to treat C2C12 myoblasts. Erastin, known as a ferroptosis inducer, was used as a positive control. The results showed that the viability of C2C12 myoblasts was negatively correlated with the concentration of FAC (Fig. 1A and B), and 500 μM FAC was chosen for further cell experiments in C2C12 myoblasts due to its similar effect to erastin. Consistent with erastin-induced ferroptosis, FAC treatment significantly increased lipid peroxidation (Fig. 1D and E) and MDA content (Fig. 1G), as well as *Ptgs2* mRNA levels (Fig. 1H), and decreased NADPH content (Fig. 1F). Iron overload-induced ferroptosis was confirmed using the specific ferroptosis inhibitors ferrostatin-1 (Ferr-1) and deferoxamine (DFO), both of which significantly reversed the changes in cell death, lipid peroxidation, Ptgs2 mRNA levels, NADPH, and MDA content (Fig. 1C-H). Like C2C12 myoblasts, C2C12 myotubes also exhibited FAC (1 mM)-induced ferroptosis, as shown by the increasing lipid peroxidation (Fig. 2B and C), MDA content (Fig. 2E), and *Ptgs2* mRNA levels (Fig. 2F), and decreasing cell viability (Fig. 2A) and NADPH content (Fig. 2D). Similarly, both the inhibitors Ferr-1 and DFO rescued the appearance of cells after treatment with FAC (Fig. 2).

### Myogenic differentiation in C2C12 cells was impaired by iron overload

**Figure 3:**
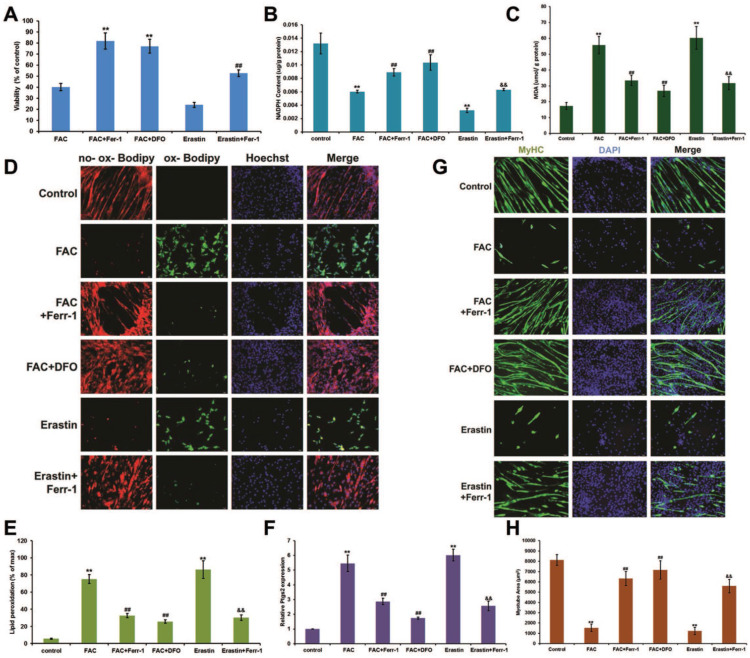
Ferroptosis impaired differentiation in C2C12 cells. Differentiating C2C12 cells were treating with 500 µM FAC or 1 µM erastin with or without inhibitors (ferr-1 or DFO) for 5 days. (**A**) Cell viability of cells after 5 days treatment.^ **^P < 0.01 versus FAC and ^##^*P* < 0.01 versus erastin. NADPH (**B**), MDA content (**C**), Lipid peroxidation (**E**) and* Ptgs2* mRNA level (**F**) in C2C12 cells after 5 days treatment. ^**^P < 0.01 versus control, ^##^*P* < 0.01 versus FAC, and^ &&^*P* < 0.01 versus erastin. (**D**) C2C12 cells stained with C11-BODIPY (red: no probe staining, and green: ox-probe staining. Original magnification: 200 x). (**G**) Immunocytochemical staining of MyHC (green) and nuclei (DAPI in blue) in C2C12 cells after 5 days treatment (Original magnification: 200 x). (**H**) Myotube area in C2C12 cells after 5 days treatment. ^**^P < 0.01 versus control, ^##^*P* < 0.01 versus FAC, and^ &&^*P* < 0.01 versus erastin.

Next, we examined the effect of iron overload on the differentiation of C2C12 myoblasts. Cells were cultured in growth medium (GM) until they reached 80% confluence, and the medium was then switched to normal differentiation medium (DM) or FAC added differentiation medium (FDM). After 5 days of treatment with FDM, we found that the cell viability (Fig. 3A) and NADPH content (Fig. 3B) significantly decreased, while the MDA content (Fig. 3C), lipid peroxidation (Fig. 3D and E), and Ptgs2 mRNA levels significantly (Fig. 3F) increased, which could all be rescued by inhibitors of ferroptosis (Ferr-1and DFO). Interestingly, the late myogenic marker, myosin heavy chain (MyHC), was not only remarkably reduced after FAC and erastin treatment, but also rescued by inhibitors of ferroptosis (Ferr-1and DFO), which suggested that the differentiation of C2C12 cells was impaired by iron overload-induced ferroptosis (Fig. 3G and H).

### Sarcopenia-like phenotype observed in senescence accelerated mouse prone 8 (SAMP8) mice

**Figure 4:**
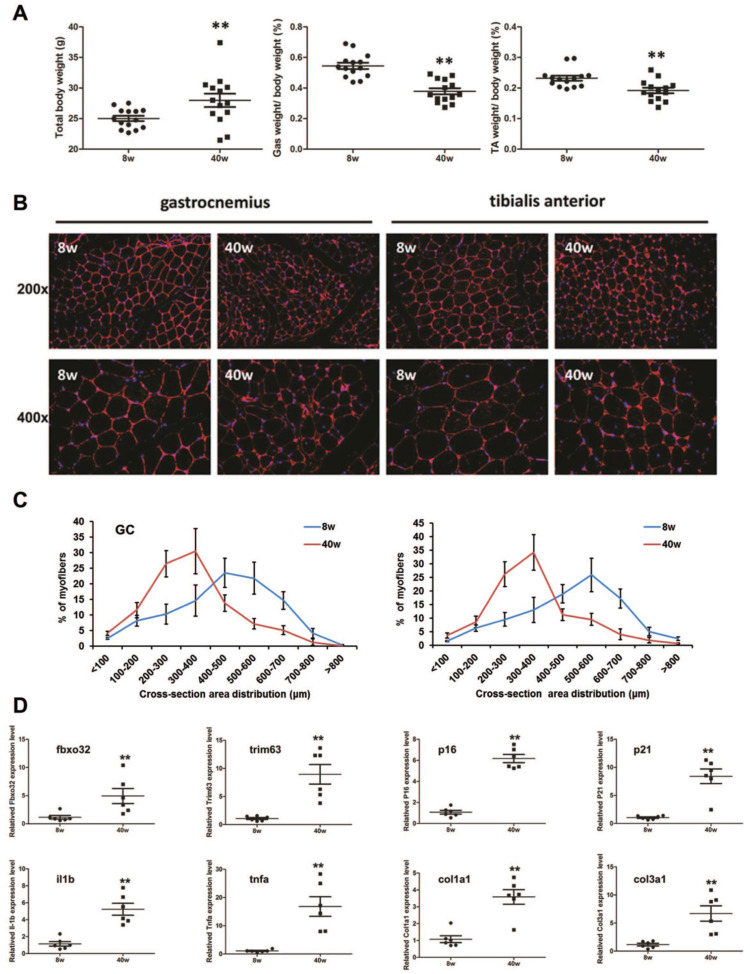
Sarcopenia-like phenotype in old SAMP8 mice. (**A**) Whole body weight and percentage of gastrocnemius (GC) or tibialis anterior (TA) muscle in whole body muscles by weight in young (8 weeks old, 8 w) and old (40 weeks old, 40 w) SAMP8 mice. ^**^P < 0.01 for young mice versus old mice. (**B**) Immunostaining for laminin (red) in cross sections of the GC or TA muscles from young and old SAMP8 mice (Original magnification: 200 x and 400 x). (**C**) Percentage distribution of muscle fiber cross-section area derived from GC or TA muscles in young and old SAMP8 mice. (**D**) Relative expression levels of muscle atrophy (*fbxo32* and* trim63*), inflammation (*il1b* and *tnfa*), fibrosis-related (*col1a1* and *col3a1*), and senescence marker (*p16* and *p21*) genes in GC and TA muscles from young and old SAMP8 mice. ^**^P < 0.01 versus young mice.

Compared with young (8-week-old) SAMP8 mice, old (40-week-old) SAMP8 mice exhibited a reduced muscle mass in the gastrocnemius muscle (GC) and tibialis anterior muscle (TA), though they showed larger body weight (Fig. 4A). We then found that the muscle fiber sizes in the old SAMP8 mice were significantly smaller than those in young SAMP8 mice (Fig. 4B and C). As muscle atrophy markers, the expression levels of Fbxo32 and Trim63 were significantly increased in the muscles of old SAMP8 mice. Consistent with this, aging markers, inflammation- and fibrosis-related genes, such as p16, p21, interleukin (IL)-1β, tumor necrosis factor (TNF)-α,and collagen types I and III, all found to be highly expressed in aged WT mice in previous studies 28, also showed upregulation in the muscles of old SAMP8 mice (Fig. 4D). Taken together, these findings indicated that old (40 weeks) SAMP8 mice exhibited a sarcopenia-like phenotype and could be used as an* in vivo* model of sarcopenia.

### Iron overload induces ferroptosis in sarcopenia mice

**Figure 5:**
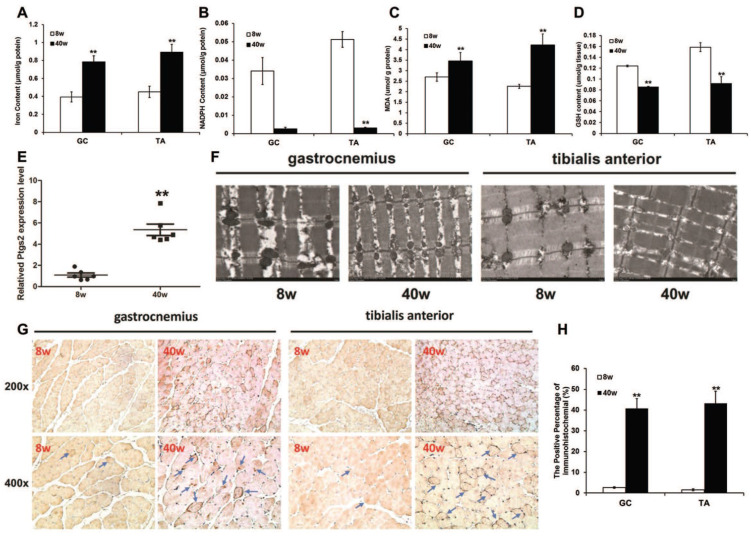
Ferroptosis in muscles of old SAMP8 mice. (**A**) Iron content, (**B**) NADPH content, (**C**) MDA content, (**D**) GSH content, and (**E**) *Ptgs2* mRNA were measured in the GC and TA muscles of young (8 weeks old, 8 w) and old (40 weeks old, 40 w) SAMP8 mice.^ *^P < 0.01 presents young mice versus old mice. (**F**) GC and TA muscles examined using transmission electron microscopy (Original magnification: 6000 x). (**G**) Muscle sections were stained with 4-Hydroxynonenal and brown indicates positive staining (Blue arrows pointed. Original magnification: 200 x and 400 x). (**H**) Quantification of immunohistochemistry. ^**^P < 0.01 versus young mice.

To investigate whether ferroptosis is associated with sarcopenia, we measured markers of ferroptosis in old SAMP8 mice. A remarkable iron overload was found in old mice. The iron content of the muscles from old SAMP8 mice was much higher than the level in young SAMP8 mice (Fig. 5A). Consistent with this increased iron content in muscles, the old SAMP8 mice had significantly lower muscular NADPH (Fig. 5B) and GSH contents (Fig. 5D), as well as a higher muscular MDA content (Fig. 5C) and* Ptgs2* mRNA expression level (Fig. 5E), compared to young SAMP8 mice. Transmission electron microscopy analysis demonstrated that ferroptosis involves the morphological features of mitochondria in the muscles of old SAMP8 mice, which displayed ruptures and were smaller than those of young SAMP8 mice (Fig. 5F). In addition to MDA, as one of the products of lipid peroxidation, we stained for 4-hydroxynonenal (4-HNE) by immunostaining. The positive staining areas were then quantified, and the data indicated that the 4-HNE levels in the muscles of old SAMP8 mice were significantly higher than those in young SAMP8 mice (Fig. 5G and H). All of the above results strongly suggested that iron overload could induce ferroptosis in the muscles of sarcopenia mice.

### P53-mediated SLC7A11activity involved in iron overload-induced ferroptosis

**Figure 6:**
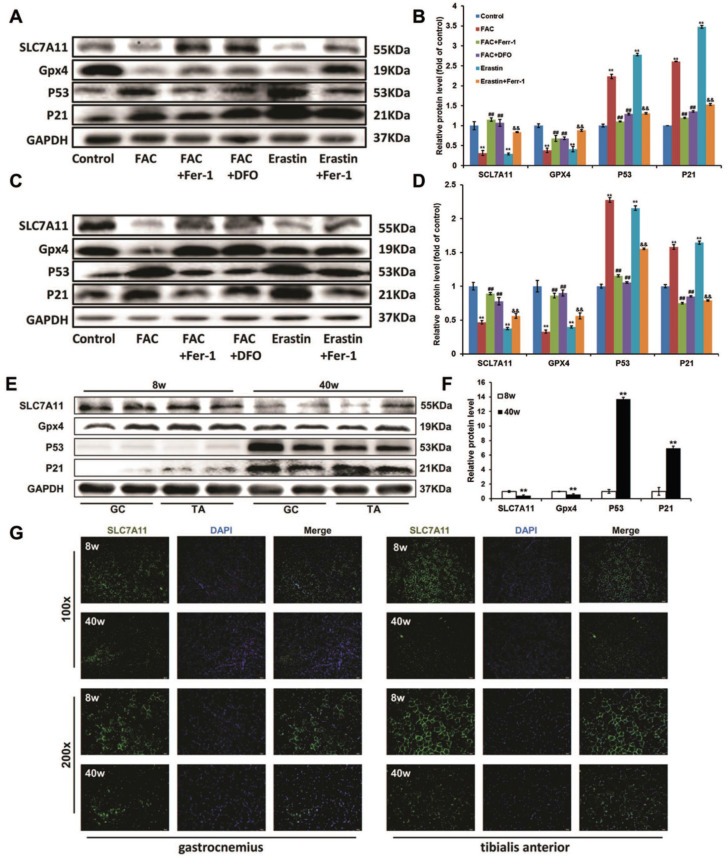
P53-mediated SLC7A11 activity in ironoverload-induced ferroptosis. Protein levels of SLC7A11, Gpx4, p53 and p21 in treated C2C12 myoblasts (**A, B**), treated C2C12 myotubes (C, D), and the GC and TA muscles of young (8 weeks old, 8 w) and old (40 weeks old, 40 w) SAMP8 mice (**E, F**). **B, D:** ^**^P < 0.01 versus control, ^##^*P* < 0.01 versus FAC, and^ &&^*P* < 0.01 versus erastin. **F:** ^**^P < 0.01 versus young mice. (**G**) Representative immunofluorescence staining of SLC7A11 (green) in the GC and TA muscles of the young (8 weeks old, 8 w) and old (40 weeks old, 40 w) SAMP8 mice. Original magnification: 100 x and 200 x.

In both C2C12 myoblasts and C2C12 myotubes (Fig. 6A-D), like erastin, FAC treatments down-regulated the expression of glutathione peroxidase 4 (*Gpx4*), which could then be reversed by Ferr-1 and DFO. SLC7A11, which encodes a component of the cystine/glutamate antiporter 15, was also found to be reduced at the protein level after FAC treatment. In view of some studies showing that SLC7A11 functioned as a novel p53 target gene 29, 30, we then checked the p53 protein level. Western blot analysis revealed that the expression p53 and its downstream actor p21 were severely increased in FAC-treated C2C12 cells, consistent with the decreased expression level of SLC7A11. All of these effects could be rescued by treatment with ferroptosis inhibitors (Ferr-1 and DFO), suggesting that p53-mediated transcriptional repression of *SLC7A11* was critical for iron overload-induced ferroptosis. Similarly, a lower expression of SLC7A11 and Gpx4 as well as a higher expression of p53 and p21 were found in the sarcopenia muscles of old SAMP8 mice, as compared with young SAMP8 mice (Fig. 6E and F). In addition, the results of immunofluorescence staining indicated that SLC7A11 was localized predominantly to the plasma membrane and showed a markedly higher level in old SAMP8 mice compared to young SAMP8 mice (Fig. 6G).

## Discussion

The C2C12 cell line is a murine myoblast cell line derived from satellite cells, which is commonly used as an* in vitro* model of muscle differentiation and regeneration, due to their ability as satellite cells to transform from myoblasts into differentiated myofibers with adequate stimulation 31. In our present study, FAC treatment was separately applied to undifferentiated C2C12 myoblasts and differentiated C2C12 myotubes, as well as cells during the process of differentiation. All cells displayed ferroptosis characteristics, such as a decrease in cell viability and NADPH content, as well as an increase in lipid peroxidation, MDA content, and *Ptgs2* mRNA levels, which have been previously demonstrated as biomarkers for the detection of ferroptosis 21, 24, 32. Interestingly, differentiated myotubes displayed a significant reduction after treatment with FAC in the process of differentiation, which might have been due to the lower viability of the treated cells. In addition, treatment with ferrostatin-1 (Ferr-1) and deferoxamine (DFO) have been, respectively, considered as specific inhibitors of ferroptosis and as an iron chelator 24, and remarkably reversed biomarkers of ferroptosis, further confirming that iron overload induces ferroptosis in muscle cells.

Aged mice are a natural choice for an animal model for sarcopenia study, but their application is hindered because of the huge cultivation time and cost required. Thus, accelerated animal models of aging afford us the convenience of shortening the requisite to wait for senescence. Senescence Accelerated Mouse (SAM) models consist of 18 different lines: 11 senescence-prone inbred strains (SAMP) and 7 senescence-resistant inbred strains (SAMR), which are regarded as a good choice for “fast-forward” observations of aging's influence 33, 34. Examinations of the properties of muscle physiology, including muscle metabolites, muscle mass, contractile properties, force generation, fatigability, and fiber size and distribution between 2 strains of senescent-accelerated (SAMP6, SAMP8) and 1 strain of senescent-resistant (SAMR1) mice, showed that SAMP8 exhibited the features most demonstrative of accelerated muscle aging. This included a greater reduction in muscle phosphorcreatine levels, muscle mass and contractility, and type-II fiber size atrophy, with aging features developing earlier and at a faster rate than SAMP6 or SAMR1 mice 26, suggesting that the SAMP8 mouse is a reasonable model for muscular aging and sarcopenia studies. Moreover, it has been reported that sarcopenia onset occurs in 8-month-old SAMP8 mice as pre-sarcopenia and develops into sarcopenia in 10-month-old SAMP8 mice, which was shown as the initial decline of muscle mass, structural and contractile properties from month 8 and the significant decrease at month 10 27, 35, 36. Therefore, in our present study, 40-week-old SAMP8 mice were chosen as models of sarcopenia, as they actually displayed decreasing muscle mass and fiber size. In addition, old SAMP8 mice showed remarkable difference from young controls in terms of the expression level of marker genes, including muscle atrophy genes (*fbxo32* and* trim63*), inflammation and fibrosis- related genes (*il1b*, *tnfa*, *col1a*, and *col3a1*), and senescence genes (*p16* and *p21*), whose patterns were similar to that in sarcopenia mice 28.

It has been reported that iron regulates some programmed cell death, such as apoptosis 37 and autophagy 38. Recently, iron overload was discovered to be involved in ferroptosis, a newfound form of cell death, which then leads to several pathological conditions, including hemochromatosis 24 and cardiomyopathy 25. Therefore, it is not surprising that ferroptosis plays a role in the muscle atrophy of sarcopenia, which has been shown to be related to excess iron. In fact, as hallmarks of ferroptosis, increasing lipid-associated radicals and lipid peroxides along with iron accumulation 15, may trigger particular cell death and induce some diseases. In our present study, lipid peroxidation was detected not only by staining with probes but also by testing of MDA content and 4-HNE levels, which are both considered as key products of lipid peroxidation 39. Additionally, selenoenzyme glutathione peroxidase 4 (Gpx4), due to its unique activity to prevent the uncontrolled peroxidation of phospholipids (PLs), has been proposed to be the most central downstream ferroptosis regulator, and was also remarkably repressed in the iron overloaded cells and muscle tissue of sarcopenia mice in this study. All of the results indicated that iron accumulation-induced ferroptosis, by increasing lipid peroxidation, could lead to the reduction of cell numbers and the inhibition of the differentiation from myoblasts to myotubes, which may be one of the causes of sarcopenia.

As one of the most commonly mutated genes in human cancer,* p53* is known to play a central role in tumor development by regulating a series of downstream targets involved in cell cycle arrest, DNA repair, and cellular senescence 40. It has been well documented that p53-mediated cellular senescence can lead to aging-related phenotypes, such as tissue atrophy, stem cell depletion, and impaired wound healing 41. Upon exposure to stress signals, the p53 pathway is activated, resulting in cellular senescence by enhancing the expression of some p53 targets, one of which is the cyclin-dependent kinase inhibitor p21 that induces G1 cell cycle arrest by inhibiting the activity of cyclin-CDK2/4 complexes, E2F, and PCNA 41. In our study, enhanced expression levels of p53 and p21 were found in muscle tissues of sarcopenia mice, which was consistent with the aging characteristics of mice. In addition, p53 is known to be upregulated upon exposure to excess iron but downregulated upon iron depletion, potentially via MDM2, suggesting that p53 is also regulated by several key regulators of iron metabolism 42. Moreover, p53 was recently found to regulate the expression of SLC7A11, a component of the cystine/glutamate antiporter system x_c_^-^, and induce ferroptosis upon reactive oxygen species (ROS)-induced stress 30. Therefore, in our study, we determined the expression level of *SLC7A11* and found a corresponding pattern to those of p53 and p21, which indicated that iron overload, regulates ferroptosis through the p53-SLC7A11 pathway. However, the regulatory mechanism of iron overload on p53 is still unclear, presumably due to ROS increase induced by iron accumulation 30, 42, which needs further investigation.

**Figure 7:**
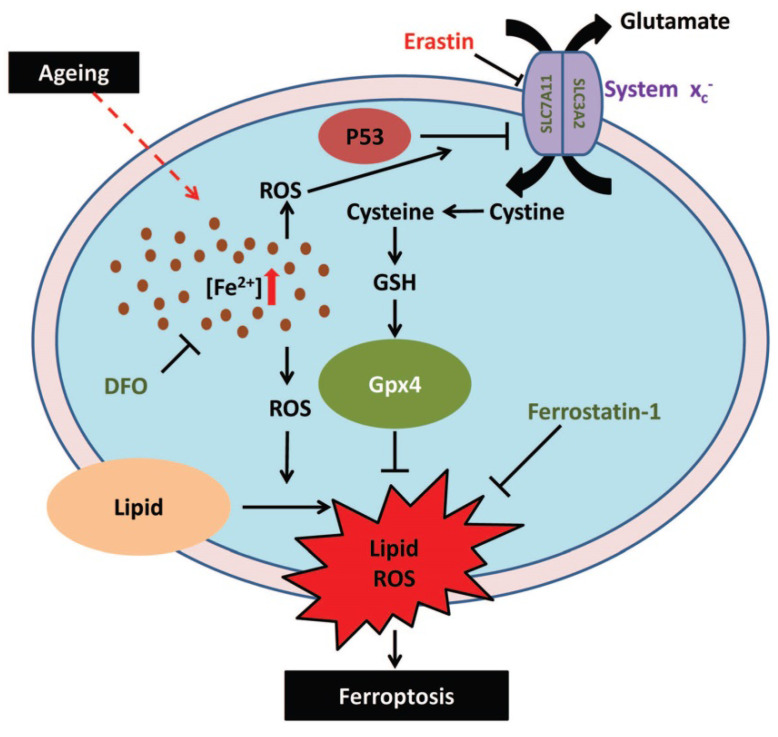
Schematic illustration of the probable regulatory mechanism of iron overload-induced ferroptosis in the muscle cells of sarcopenia mice.

In summary, in this study, we demonstrated that iron overload-induced ferroptosis plays an essential role in sarcopenia, and may be driven by the iron-p53-SLC7A11 signaling network from a mechanistic perspective (Fig. 7). Our findings shed new light on the pathogenesis of sarcopenia, which may provide some therapeutic strategies for targeting iron accumulation or ferroptosis to treat sarcopenia in the elderly.

## References

[1] Anker SD, Morley JE, von Haehling S (2016). Welcome to the ICD-10 code for sarcopenia. Journal of cachexia, sarcopenia and muscle.

[2] Morley JE, Baumgartner RN, Roubenoff R, Mayer J, Nair KS (2001). Sarcopenia. The Journal of laboratory and clinical medicine.

[3] Liu CK, Leng X, Hsu FC, Kritchevsky SB, Ding J, Earnest CP (2014). The impact of sarcopenia on a physical activity intervention: the Lifestyle Interventions and Independence for Elders Pilot Study (LIFE-P). The journal of nutrition, health & aging.

[4] Janssen I, Shepard DS, Katzmarzyk PT, Roubenoff R (2004). The healthcare costs of sarcopenia in the United States. Journal of the American Geriatrics Society.

[5] Marzetti E, Calvani R, Tosato M, Cesari M, Di Bari M, Cherubini A (2017). Sarcopenia: an overview. Aging clinical and experimental research.

[6] Greenlund LJ, Nair KS (2003). Sarcopenia-consequences, mechanisms, and potential therapies. Mechanisms of ageing and development.

[7] Brass EP, Sietsema KE (2011). Considerations in the development of drugs to treat sarcopenia. Journal of the American Geriatrics Society.

[8] Sheftel AD, Mason AB, Ponka P (2012). The long history of iron in the Universe and in health and disease. Biochimica et biophysica acta.

[9] Mena NP, Urrutia PJ, Lourido F, Carrasco CM, Nunez MT (2015). Mitochondrial iron homeostasis and its dysfunctions in neurodegenerative disorders. Mitochondrion.

[10] Zhao G (2018). Is Iron Accumulation a Possible Risk Factor for Sarcopenia?. Biological trace element research.

[11] Ikeda Y, Imao M, Satoh A, Watanabe H, Hamano H, Horinouchi Y (2016). Iron-induced skeletal muscle atrophy involves an Akt-forkhead box O3-E3 ubiquitin ligase-dependent pathway. Journal of trace elements in medicine and biology: organ of the Society for Minerals and Trace Elements.

[12] Altun M, Edstrom E, Spooner E, Flores-Moralez A, Bergman E, Tollet-Egnell P (2007). Iron load and redox stress in skeletal muscle of aged rats. Muscle & nerve.

[13] DeRuisseau KC, Park YM, DeRuisseau LR, Cowley PM, Fazen CH, Doyle RP (2013). Aging-related changes in the iron status of skeletal muscle. Experimental gerontology.

[14] Aydemir TB, Troche C, Kim J, Kim MH, Teran OY, Leeuwenburgh C (2016). Aging amplifies multiple phenotypic defects in mice with zinc transporter Zip14 (Slc39a14) deletion. Experimental gerontology.

[15] Dixon SJ, Lemberg KM, Lamprecht MR, Skouta R, Zaitsev EM, Gleason CE (2012). Ferroptosis: an iron-dependent form of nonapoptotic cell death. Cell.

[16] Dixon SJ, Stockwell BR (2014). The role of iron and reactive oxygen species in cell death. Nature chemical biology.

[17] Yang WS, Kim KJ, Gaschler MM, Patel M, Shchepinov MS, Stockwell BR (2016). Peroxidation of polyunsaturated fatty acids by lipoxygenases drives ferroptosis. Proceedings of the National Academy of Sciences of the United States of America.

[18] Friedmann Angeli JP, Schneider M, Proneth B, Tyurina YY, Tyurin VA, Hammond VJ (2014). Inactivation of the ferroptosis regulator Gpx4 triggers acute renal failure in mice. Nature cell biology.

[19] Yang WS, Stockwell BR (2016). Ferroptosis: Death by Lipid Peroxidation. Trends in cell biology.

[20] Xie Y, Hou W, Song X, Yu Y, Huang J, Sun X (2016). Ferroptosis: process and function. Cell death and differentiation.

[21] Yang WS, SriRamaratnam R, Welsch ME, Shimada K, Skouta R, Viswanathan VS (2014). Regulation of ferroptotic cancer cell death by GPX4. Cell.

[22] Skouta R, Dixon SJ, Wang J, Dunn DE, Orman M, Shimada K (2014). Ferrostatins inhibit oxidative lipid damage and cell death in diverse disease models. Journal of the American Chemical Society.

[23] Linkermann A, Skouta R, Himmerkus N, Mulay SR, Dewitz C, De Zen F (2014). Synchronized renal tubular cell death involves ferroptosis. Proceedings of the National Academy of Sciences of the United States of America.

[24] Wang H, An P, Xie E, Wu Q, Fang X, Gao H (2017). Characterization of ferroptosis in murine models of hemochromatosis. Hepatology.

[25] Fang X, Wang H, Han D, Xie E, Yang X, Wei J (2019). Ferroptosis as a target for protection against cardiomyopathy. Proceedings of the National Academy of Sciences of the United States of America.

[26] Derave W, Eijnde BO, Ramaekers M, Hespel P (2005). Soleus muscles of SAMP8 mice provide an accelerated model of skeletal muscle senescence. Experimental gerontology.

[27] Guo AY, Leung KS, Siu PM, Qin JH, Chow SK, Qin L (2015). Muscle mass, structural and functional investigations of senescence-accelerated mouse P8 (SAMP8). Experimental animals.

[28] Yoshida N, Endo J, Kinouchi K, Kitakata H, Moriyama H, Kataoka M (2019). (Pro)renin receptor accelerates development of sarcopenia via activation of Wnt/YAP signaling axis. Aging cell.

[29] Lachaier E, Louandre C, Godin C, Saidak Z, Baert M, Diouf M (2014). Sorafenib induces ferroptosis in human cancer cell lines originating from different solid tumors. Anticancer research.

[30] Jiang L, Kon N, Li T, Wang SJ, Su T, Hibshoosh H (2015). Ferroptosis as a p53-mediated activity during tumour suppression. Nature.

[31] Yaffe D, Saxel O (1977). Serial passaging and differentiation of myogenic cells isolated from dystrophic mouse muscle. Nature.

[32] Shimada K, Hayano M, Pagano NC, Stockwell BR (2016). Cell-Line Selectivity Improves the Predictive Power of Pharmacogenomic Analyses and Helps Identify NADPH as Biomarker for Ferroptosis Sensitivity. Cell chemical biology.

[33] Romanick M, Thompson LV, Brown-Borg HM (2013). Murine models of atrophy, cachexia, and sarcopenia in skeletal muscle. Biochimica et biophysica acta.

[34] Moorwood C, Liu M, Tian Z, Barton ER Isometric and eccentric force generation assessment of skeletal muscles isolated from murine models of muscular dystrophies. Journal of visualized experiments: JoVE. 2013: e50036.

[35] Zhang N, Chow SKH, Leung KS, Lee HH, Cheung WH (2017). An animal model of co-existing sarcopenia and osteoporotic fracture in senescence accelerated mouse prone 8 (SAMP8). Experimental gerontology.

[36] Zhang N, Chim YN, Wang J, Wong RMY, Chow SKH, Cheung WH (2020). Impaired Fracture Healing in Sarco-Osteoporotic Mice Can Be Rescued by Vibration Treatment Through Myostatin Suppression. Journal of orthopaedic research: official publication of the Orthopaedic Research Society.

[37] Wang GS, Eriksson LC, Xia L, Olsson J, Stal P (1999). Dietary iron overload inhibits carbon tetrachloride-induced promotion in chemical hepatocarcinogenesis: effects on cell proliferation, apoptosis, and antioxidation. Journal of hepatology.

[38] Lunova M, Goehring C, Kuscuoglu D, Mueller K, Chen Y, Walther P (2014). Hepcidin knockout mice fed with iron-rich diet develop chronic liver injury and liver fibrosis due to lysosomal iron overload. Journal of hepatology.

[39] Yamada N, Karasawa T, Kimura H, Watanabe S, Komada T, Kamata R (2020). Ferroptosis driven by radical oxidation of n-6 polyunsaturated fatty acids mediates acetaminophen-induced acute liver failure. Cell death & disease.

[40] Harms K, Nozell S, Chen X (2004). The common and distinct target genes of the p53 family transcription factors. Cellular and molecular life sciences: CMLS.

[41] Qian Y, Chen X (2013). Senescence regulation by the p53 protein family. Methods in molecular biology.

[42] Zhang J, Chen X (2019). p53 tumor suppressor and iron homeostasis. The FEBS journal.

